# Bovine herpesvirus 1 tegument protein *UL21* plays critical roles in viral secondary envelopment and cell-to-cell spreading

**DOI:** 10.18632/oncotarget.21776

**Published:** 2017-10-10

**Authors:** Farzana Shahin, Sohail Raza, Kui Yang, Changmin Hu, Yingyu Chen, Huanchun Chen, Aizhen Guo

**Affiliations:** ^1^ The State Key Laboratory of Agricultural Microbiology, Huazhong Agricultural University, Wuhan 430070, China; ^2^ College of Veterinary Medicine, Huazhong Agricultural University, Wuhan 430070, China; ^3^ Department of Pathobiological Sciences, School of Veterinary Medicine, Louisiana State University, Baton Rouge, Louisiana 70803, USA; ^4^ Key Laboratory of Development of Veterinary Diagnostic Products, Ministry of Agriculture, Wuhan 430070, China; ^5^ Hubei International Scientific and Technological Cooperation Base of Veterinary Epidemiology, Huazhong Agricultural University, Wuhan 430070, China; ^6^ Department of Microbiology, University of Veterinary and Animal Sciences, Lahore 54000, Pakistan

**Keywords:** BoHV-1, interacting proteins, secondary envelopment, tegument, UL21

## Abstract

*Bovine herpesvirus 1* (BoHV-1) *UL21* is a tegument protein thought to be indispensable for efficient viral growth but its precise function in BoHV-1 is currently unknown. To determine the function of *UL21* in BoHV-1 replication, we constructed a mutant virus bearing a *UL21* deletion (vBoHV-1-∆UL21) and its revertant virus, vBoHV-1-∆UL21R, in which the *UL21* gene was restored using a bacterial artificial chromosome system. The replication of vBoHV-1-∆UL21 was 1,000-fold lower and its plaque size was 85% smaller than those of the wild-type virus (BoHV-1). An ultrastructural analysis showed that deletion of *UL21* led to an un-enveloped capsid accumulation in the cytoplasm, whereas nucleocapsid egress was not impaired, suggesting that *UL21* is critical for secondary envelopment in BoHV-1. Co-immunoprecipitation assays revealed that HA-tagged *UL21* pulled down *UL16*, suggesting that these two proteins form a complex, and this was further confirmed by a co-immunofluorescence assay. Taken together, these data provide evidence that *UL21* plays critical roles in BoHV-1 secondary envelopment, and *UL16* is likely to be involved in these activities.

## INTRODUCTION

*Bovine herpesvirus 1* (BoHV-1), an enveloped double-stranded-DNA virus that belongs to the subfamily *Alphaherpesvirinae* of the *Herpesvirida*e family, causes bovine respiratory disease, vulvovaginitis, abortion, conjunctivitis, balanoposthitis, and severe neonatal disease in cattle [1]. It is divided into three sub-genotypes: BoHV-1.1, BoHV-1.2a, and BoHV-1.2b, which can be differentiated by genomic sequencing and restriction fragment length polymorphisms (RFLPs) [2, 3]. BoHV-1 infects cattle of all ages and breeds worldwide, and it causes severe economic losses to the cattle industry [4]. The morphogenesis and maturation of viral particles is an important stage of viral replication, which contributes to the pathogenesis of herpesviruses. The mature BoHV-1 virion consists of a multilayered architecture that is common in all herpesviruses: a capsid enclosing the double-stranded DNA genome, an envelope, and the tegument. Tegument is a cluster of proteins that occupy the space between the envelope and nucleocapsids. All alpha herpesviruses possess more than 15 tegument proteins [5]. As components of viruses, tegument proteins are delivered into host cells upon viral infection, where some paly roles in the early stage of viral particle production by regulating the expression of viral immediate-early genes, as well as in the late stage of infection via their involvement in viral morphogenesis, assembly, and egress [6, 7]. These proteins play critical roles in each step of envelopment, de-envelopment, and re-envelopment process [5, 8].

*UL21* is conserved, multifunctional tegument protein in *alphaherpesviruses* [9, 10], with sequence similarity ranging between 57 and 94% at the nucleotide level and sequence identity ranging between 27 and 84% at the amino acid level. It has different essential, albeit poorly understood, functions in viral replication and pathogenesis. Previously the crystal structures of *UL21* have been reported in HSV-1 [9, 11]. The functions of *UL21* have been well-characterized in herpes simplex virus (HSV) and pseudorabies virus (PRV). Previous studies highlighted that a lack of *UL21* resulted in decreased viral titers in HSV-1 [12] and PRV [13, 14], and a *UL21* deletion mutant caused delay in production of viral mRNA, viral proteins, and virions in HSV-2 and HSV-1 [10, 12, 15]. In HSV-2 ∆UL21-infected cells, nuclear egress was blocked, which reduced viral titer to undetectable levels [10]. In HSV-1 ∆UL21 mutant showed a reduction of *UL16* in the tegument of extracellular virions [16], while a PRV ∆*UL21* mutant incorporated lower amounts of the tegument proteins UL46 and UL49, as well as a lower level of US3 into mature virions [17]. Biochemical data suggest that *UL21* gene expression in PRV facilitates viral DNA processing and is directly linked with capsid maturation [18]. Moreover, *UL21* was shown to be a capsid-associated tegument protein in HSV-1 [19] and PRV [18] via an interaction with *UL16* [14, 20], which connects the capsid to the envelope to promote secondary envelopment [21, 22]. In HSV-1, *UL21* is critical for the function of glycoprotein E by cooperating with the interacting proteins *UL11* and *UL16*. Additionally, in HSV-1, *UL21* promotes intracellular viral movement by interacting with microtubules [19]. The cytoplasmic and nuclear localization of *UL21* has been observed in HSV-1 [19, 20], particularly to the nuclear rim in HSV-1 and HSV-2 [10, 23].

Herpesvirus assembly and egress is a complex and dynamic process that requires many protein interactions and so are the roles of tegument proteins. As in HSV and PRV, *UL16* interacts with *UL11* and *UL21*, and it assists the cellular release of virions via budding and virion morphogenesis [14, 20, 24]. The teguments proteins are compositionally differ among members of the herpesvirus family. The major constituents of the BoHV-1 envelope proteins have been well-characterized [4]; however, the tegument and capsid proteins have not been studied extensively [5]. Nevertheless, the phenotype of UL21 protein in BoHV-1 has not been characterized in cell culture.

The present study was conducted to determine the functions of *UL21* in BoHV-1. To this end, we generated a mutant virus with a *UL21* deletion that does not express *pUL21* as well as its revertant. We linked an epitope tag to the minor capsid protein *UL35* in *UL21* deletion and intact *UL21* to analyze the capsid egress pathway. We found that the Δ*UL21* mutant exhibited unenveloped capsid accumulation and that its replication was approximately 1,000-fold lower than that of the wild-type virus. In addition, we found that *UL21* formed a complex with *UL16*. While the *UL16* interaction has been reported in other herpesviruses, this is the first report of an interaction between *UL21* and *UL16* in BoHV-1.

## RESULTS

### Construction and initial characterization of BoHV-1 mutant viruses

**Figure 1:**
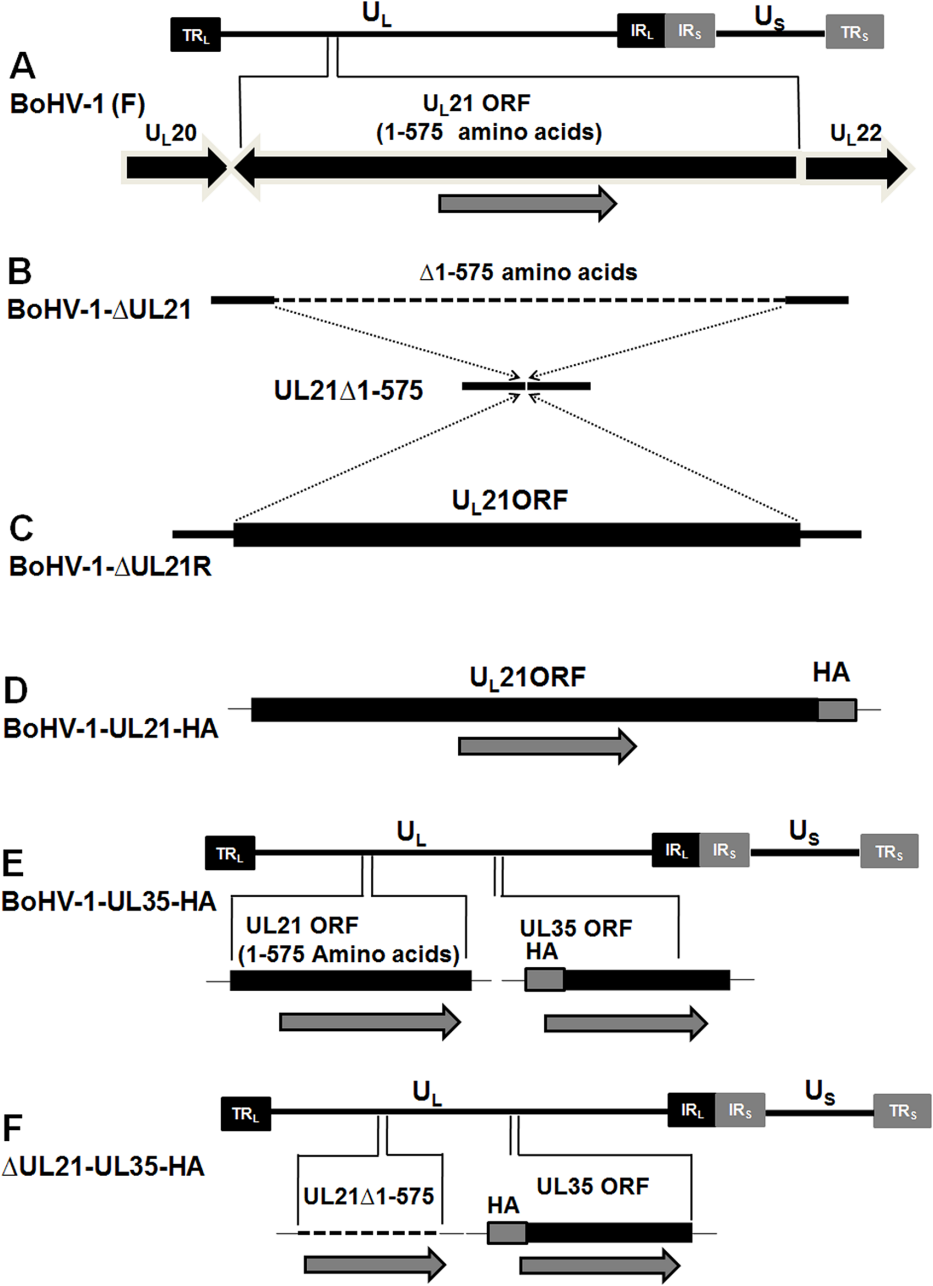
Construction and characterization of the Δ*UL21* mutant and revertant viruses **(A)** Schematic diagram showing the BoHV-1 genome (∼140 kbp) with unique short (US) and unique long (UL) regions, a terminal repeat (TR), and an internal repeat (IR). The region of the genome encoding *UL21* (codons 1-575) is located between the *UL20* and *UL22* genes. **(B)** Dashed lines show the deletion of the entire *UL21* gene from the BoHV-1 genome. **(C)** Reversion of the *UL21* gene by restoring the complete *UL21* ORF to its original location in the pBoHV-1-ΔUL21 BAC. **(D)** The insertion of a sequence encoding an HA tag at the 3′ end of the *UL21* gene in the pBoHV-1 BAC. **(E)** The insertion of a sequence encoding an HA tag at the 3′ end of the *UL35* gene in the pBoHV-1 BAC. **(F)** The addition of a sequence encoding an HA tag at the 3′ end of the *UL35* gene in the pBoHV-1-ΔUL21 BAC.

**Figure 2:**
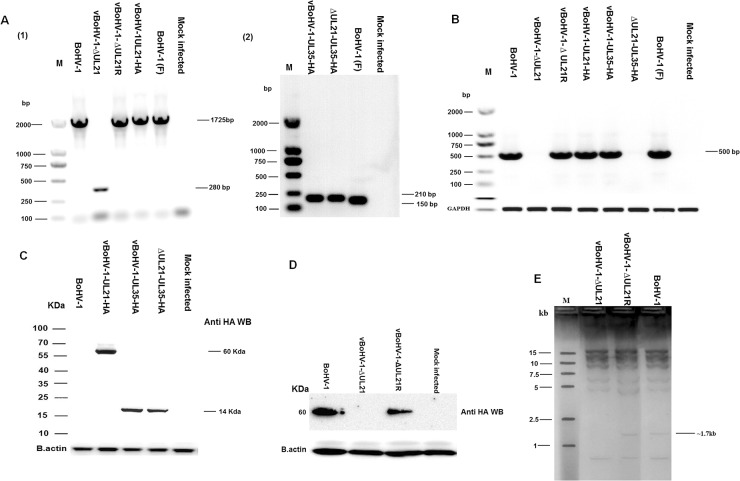
Confirmation of mutant viruses **(A)** MDBK cells were infected with each virus at a MOI of 3. PCR were run with the indicated viral DNAs of pUL21 using the UL21 F/R primers (Table 1) and pUL35 using the UL35 F/R primers (Table 1). **(B)** Expression of pUL21 recombinant viruses was confirmed by RT-PCR with the indicated viral cDNA using primers UL21 F/R (Table 1) via agarose gel electrophoresis. The bovine glyceraldehyde 3-phosphate dehydrogenase (GAPDH) gene was used as the internal reference control. DNA ladders are shown in bp to the left of the gel. **(C)** MDBK cells were infected with indicated viruses at a MOI of 3. Cells were harvested at 18 hpi and separated on a 15% SDS-PAGE and transferred to a PVDF membrane. Then, the membranes were probed sequentially with a mouse anti-HA antibody, followed by HRP-conjugated goat anti-mouse IgG. Beta-actin was used as the internal reference control. **(D)** MDBK cells were infected at a MOI of 3 with the indicated viruses. At 18 hpi, cell lysates were electrophoresed through 12% polyacrylamide gels and transferred onto PVDF membranes. Membranes were probed with the *UL21* antisera. The molecular weights of the protein markers are shown in kDa to the left of the gel. **(E)** RFLP analysis obtained by restriction enzyme *Hind*III genomic digestion of UL21 mutant, vBoHV-1-∆UL21R and BoHV-1 viruses.

A complete schematic map of the *UL21* gene encoding the 575-amino-acid *UL21* protein in the BoHV-1 genome is shown in Figure 1A. We constructed a series of recombinant viruses (v), including the *UL21* mutant vBoHV-1-ΔUL21 by deleting the entire *UL21* coding sequence (Figure 1B), as well as the revertant virus vBoHV-1-ΔUL21R (Figure 1C), the UL21 HA-tagged virus vBoHV-1-UL21-HA (Figure 1D), the UL35 HA-tagged virus vBoHV-1-UL35-HA (Figure 1E), and its *UL21* mutant vBoHV-1-ΔUL21-UL35-HA (Figure 1F). Viral genomic DNA was extracted from the vBoHV-1-∆UL21, vBoHV-1-∆UL21R, vBoHV-1-UL21-HA, vBoHV-1-UL35-HA, vBoHV-1-∆UL21-UL35-HA, and wild-type viruses. The *UL21* deletion, the reversion of this mutation, and the addition of HA tags to the *UL21* or *UL35* genes was confirmed by PCR (Figure 2A) and DNA sequencing of the PCR products. The *UL21* mRNA level was determined by RT-PCR for all the viruses. A high level of *UL21* mRNA expression was observed in viruses with either an intact *UL21* gene (wild type BoHV-1) or the revertant *UL21* gene (vBoHV-1-ΔUL21R), while *UL21* mRNA was barely detected in the Δ*UL21* mutant (vBoHV-1-ΔUL21) and the HA-tagged Δ*UL21* mutant (vBoHV-1-ΔUL21-UL35-HA) viruses as shown in Figure 2B. In addition, the expression of the HA-tagged UL21 or UL35 in the vBoHV-1-UL21-HA, vBoHV-1-UL35-HA, vBoHV-1-∆UL21-UL35-HA, and BoHV-1 (wild-type) viruses was detected by Western blotting. As expected, all the HA-tagged proteins were expressed successfully in lysates of infected Mardin-Darby bovine kidney (MDBK) cells, but not in BoHV-1 (wild type) virus and the mock-infected cells (Figure 2C).

To confirm the deletion of *UL21* in the vBoHV-1-∆UL21 virus, Western blotting assay was performed. As shown in Figure 2, panel D, *UL21* (approximately 60 kDa) was detected by antiserum against *UL21* from lysates of BoHV-1 or vBoHV-1-∆UL21R- infected MDBK cells, but not from lysates of mock or vBoHV-1-∆UL21-infected cells.

In addition, a restriction fragment length polymorphism (RFLP) analysis was performed to further confirm the *UL21* mutation (deletion). According to the results following the digestion of genomic DNA with *Hind*III, a fragment of approximately 1.7 kb was missed in the *UL21* mutant, compared with the vBoHV-1-∆UL21R and BoHV-1 (Figure 2E).

### Growth defect of the vBoHV-1∆UL21 mutant

**Figure 3:**
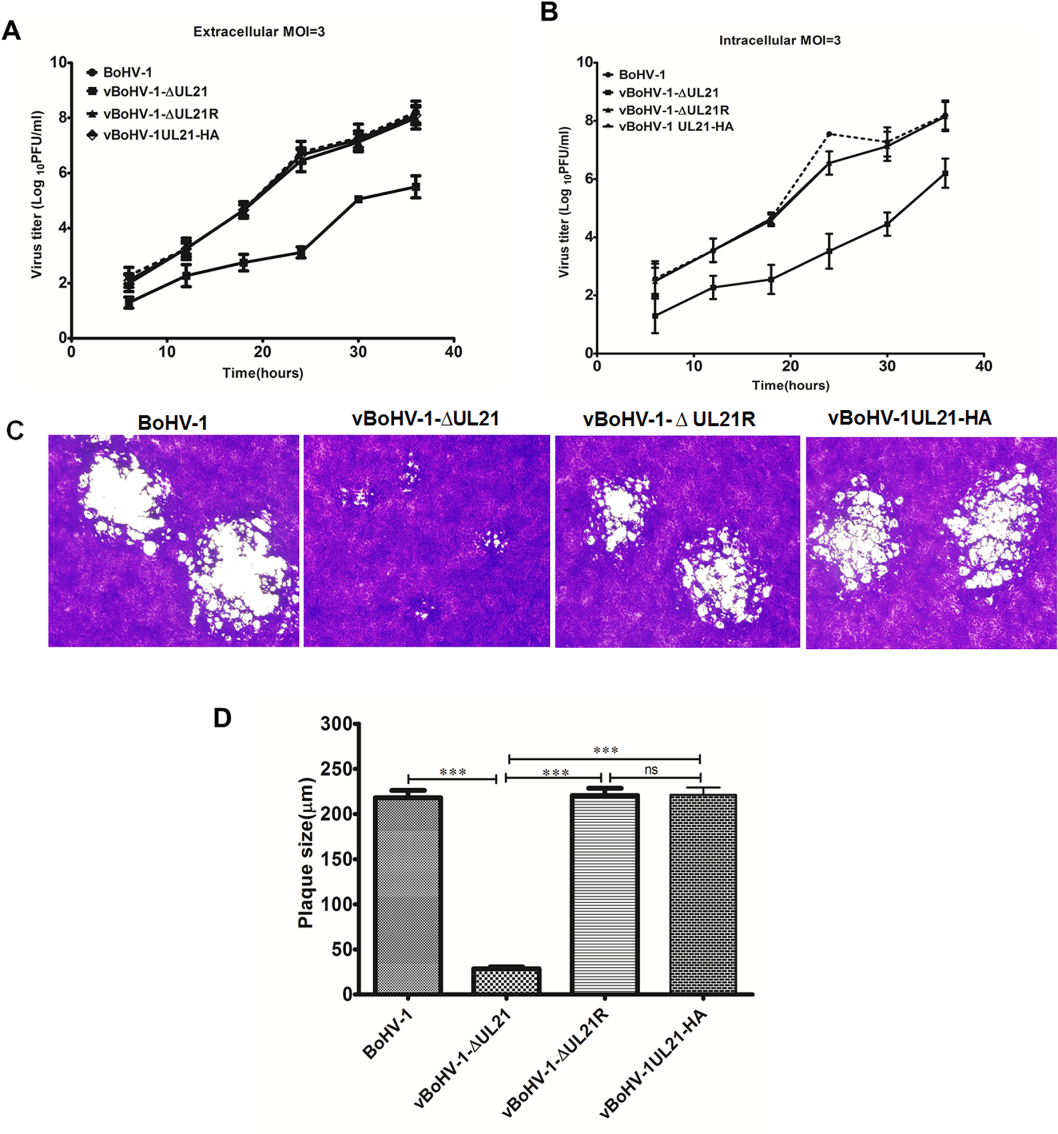
Growth analysis of the BoHV-1 recombinant viruses **(A)** Growth of the BoHV-1, vBoHV-1-∆UL21, vBoHV-1-∆UL21R and vBoHV-1UL21-HA viruses. For the single-step replication analysis, MDBK cells were infected with the indicated viruses at a MOI of 3, and harvested at 6, 12, 18, 24, 30, and 48 hpi. A *t*-test of the data revealed very significant differences between the BoHV-1 and vBoHV-1-∆UL21 viruses in terms of their extracellular (^**^, *p* < 0.01) and intracellular (^**^, *p* < 0.01) viral titers, while no significant (ns) difference was observed between the BoHV-1, vBoHV-1-∆UL21R and vBoHV-1UL21-HA viruses (*p* > 0.05). **(B)** Plaque size and morphology of the BoHV-1, vBoHV-1-∆UL21, vBoHV1-∆UL21R and vBoHV-1UL21-HA viruses. MDBK cells were infected with serial dilutions of each virus in 24-well plates and overlaid with methylcellulose. At 48 hpi, plaques were fixed with formaldehyde and stained with crystal violet. **(C)** The mean plaque sizes of the aforementioned viruses were determined with an Olympus IX70^**®**^ Microscope. The difference in the mean plaque size of the vBoHV-1-∆*UL21* virus was shown to be significantly smaller using a *t*-test (^***^, *p* < 0.001). Each independent experiment was performed three times.

To determine whether the *UL21* deletion affects BoHV-1 replication, the growth kinetics of the vBoHV-1∆UL21 virus was determined in MDBK cells. First, we compared the growth kinetics of the BoHV-1, vBoHV-1-∆UL21, vBoHV-1-∆UL21R and vBoHV-1-UL21-HA viruses using plaque assays. The results demonstrated that the replication of the vBoHV-1-∆UL21 virus was significantly lower than that of the BoHV-1, vBoHV-1-∆UL21R and vBoHV-1-UL21-HA viruses at 48 hpi. Interestingly, the extracellular titer of the vBoHV-1-∆UL21 virus was 1,000-folds lower (*p* < 0.01) than that of the BoHV-1(wild-type), vBoHV-1-∆UL21R or vBoHV-1-UL21-HA viruses (Figure 3A), while the intracellular titer of the vBoHV-1-∆UL21 virus was only 100-folds (*p* < 0.01) lower (Figure 3B) than that of the BoHV-1, vBoHV-1-∆UL21R and vBoHV-1-UL21-HA viruses. In contrast, the titers of the BoHV-1, vBoHV-1-∆UL21R and vBoHV-1-UL21-HA viruses were similar (*p* > 0.05) (Figure 3A and 3B).

To determine the effect of the *UL21* deletion on cell-to-cell spreading, the plaque size of the vBoHV-1-∆UL21 virus was compared to that of the BoHV-1, vBoHV-1-∆UL21R and vBoHV-1-UL21-HA viruses, and the diameters of 50 plaques for each virus were measured at 48 hpi. The plaques produced by the vBoHV-1-∆UL21 virus in MDBK cells were 85% smaller (*p* < 0.01) (Figure 3C) than those produced by the BoHV-1, vBoHV-1-∆UL21R or vBoHV-1-UL21-HA viruses, while the plaque sizes of the BoHV-1, vBoHV-1-∆UL21R and vBoHV-1-UL21-HA viruses did not differ significantly (*p* > 0.05) (Figure 3D). These data suggest that the HA tag did not interfere with UL21 function and furthermore, *UL21* is critical for BoHV-1 replication and cell-to-cell spreading.

### Localization of BoHV-1 UL21

**Figure 4:**
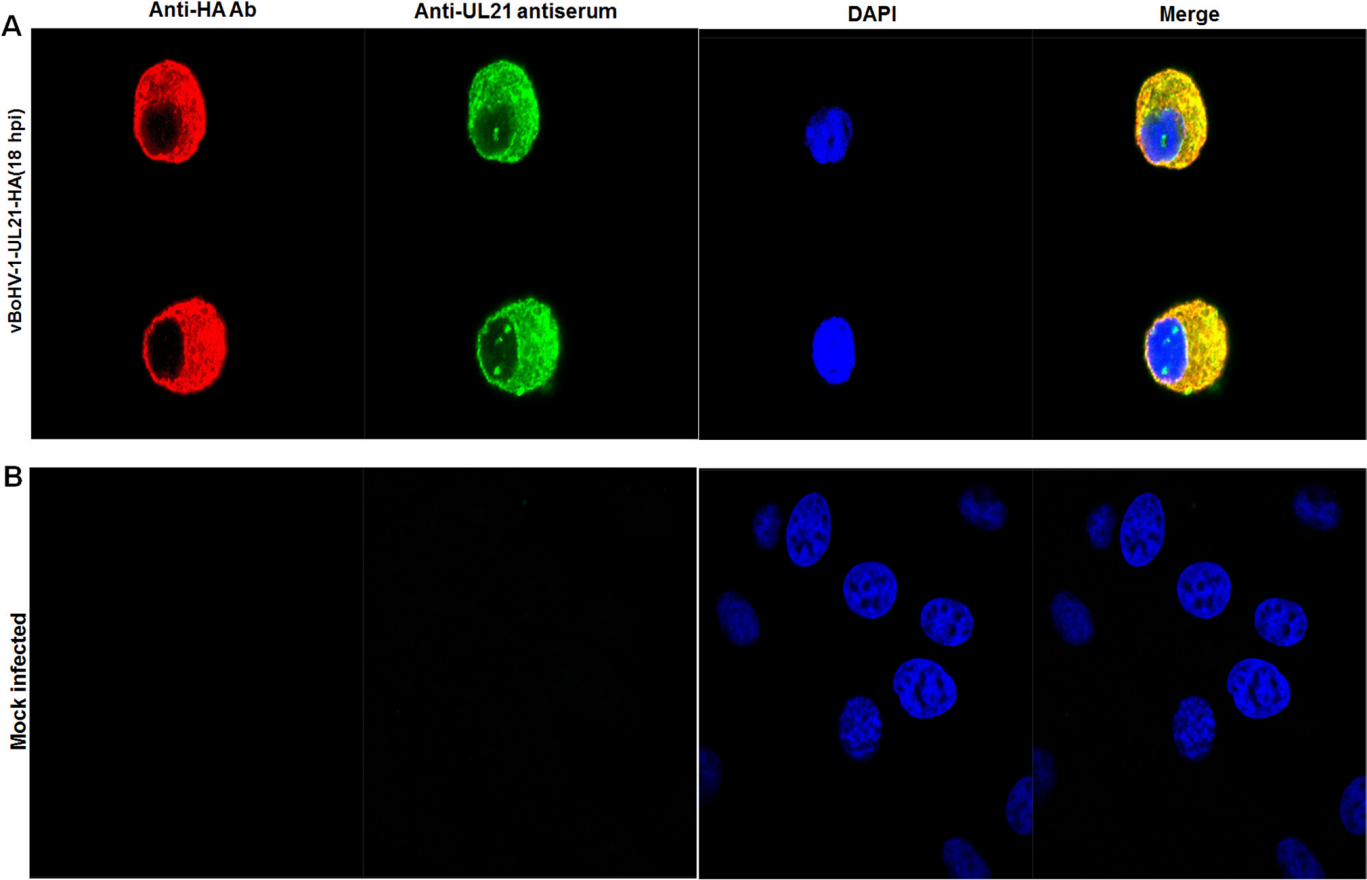
Localization of *UL21* using confocal microscopy **(A)** MDBK cells were infected with the vBoHV-1-UL21-HA virus at a MOI of 3 for 18 hrs. The infected cells were incubated with primary antibodies (mouse anti-HA) and rabbit polyclonal antisera, and then stained with Cy3-conjugated goat anti-mouse and fluorescein isothiocyanate-conjugated goat anti-rabbit IgG antibodies, Nuclei were stained blue with DAPI. Then, mount with anti-fade mounting medium. Specific capsid fluorescence was visualized under a Zeiss LSM 880 laser-scanning confocal microscope **(B)** mock-infected cells.

To gain insight into the function of BoHV-1 UL21 *in situ*, we investigated the subcellular localization of *UL21* using an indirect immunofluorescence assay. MDBK cells were mock infected or infected with the vBoHV-1-UL21-HA virus at a MOI of 3, and fluorescence was observed at 18 hpi using confocal microscopy. At 18 hpi the vBoHV-1-UL21-HA virus reacted with both the *UL21* antiserum and the anti-HA-tag antibody, and specific fluorescence was found to localize predominantly in the cytoplasm. Surprisingly, punctate nuclear staining was observed with the *UL21* antiserum, whereas nuclear staining with the anti-HA-tag antibody was barely observed. Thus, it is possible that only the *UL21* epitopes recognized by the anti-UL21 antiserum were accessible on the surface of UL21 protein in the nucleus, while the HA epitope tag was not surface exposed on the HA-tagged *UL21* fusion protein in the nucleus (Figures 4A). As expected, in mock-infected cells, which served as internal controls, no specific fluorescence was observed (Figure 4B).

### Effect of the *UL21* deletion on capsid egress

**Figure 5:**
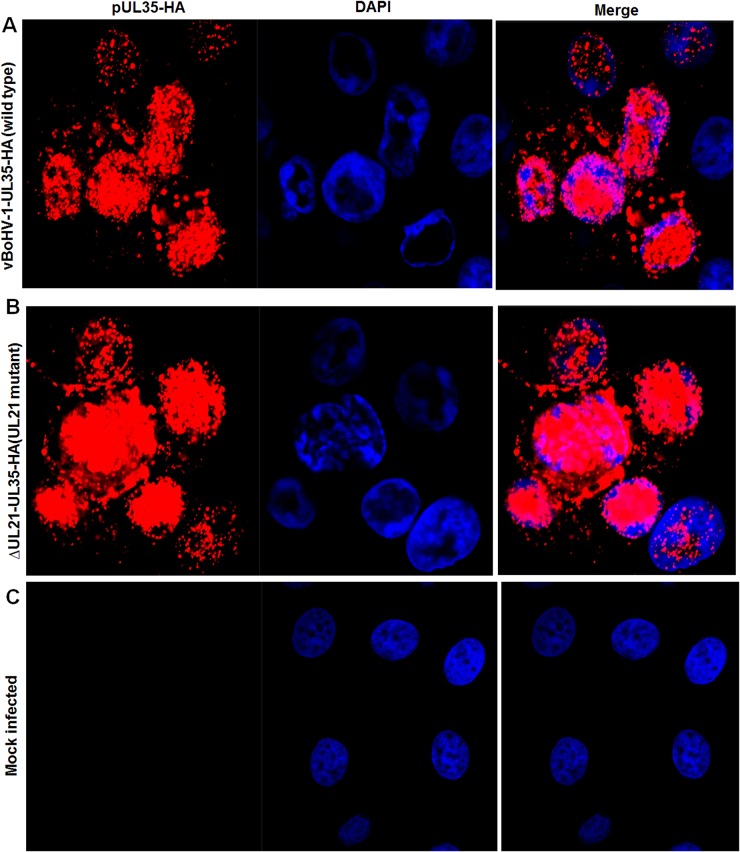
Effect of the *UL21* deletion on the capsid egress pathway MDBK cells were infected with the indicated viruses expressing HA-tagged UL35 at a MOI of 3. At 18 hpi, the cells were fixed and stained with a mouse anti-HA antibody and Cy3-conjugated goat anti-mouse IgG. The nuclei were stained blue with DAPI. Fluorescence was observed under a Zeiss LSM 880 laser-scanning confocal microscope. **(A)** Cells infected with the vBoHV-1-UL35-HA virus, **(B)** the vBoHV-1-ΔUL21-UL35-HA virus, as well as **(C)** mock-infected cells.

To investigate the role of *UL21* in nucleocapsids egress, an HA tag was attached to the carboxyl-terminus of the minor capsid protein *UL35* in the *UL21* mutant (vBoHV-1-∆UL21-UL35-HA) and wild-type (vBoHV-1-UL35-HA) viruses. At 18 hpi, punctate and high-density, concentrated fluorescence was readily detected in the cytoplasm and nuclei of both wild-type and *UL21* mutant infected cells (Figure 5A, 5B). While no specific fluorescence was observed in mock-infected cells, which served as internal controls, (Figure 5C). These data suggested that nucleocapsid egress was not impaired by *UL21* deletion.

**Figure 6:**
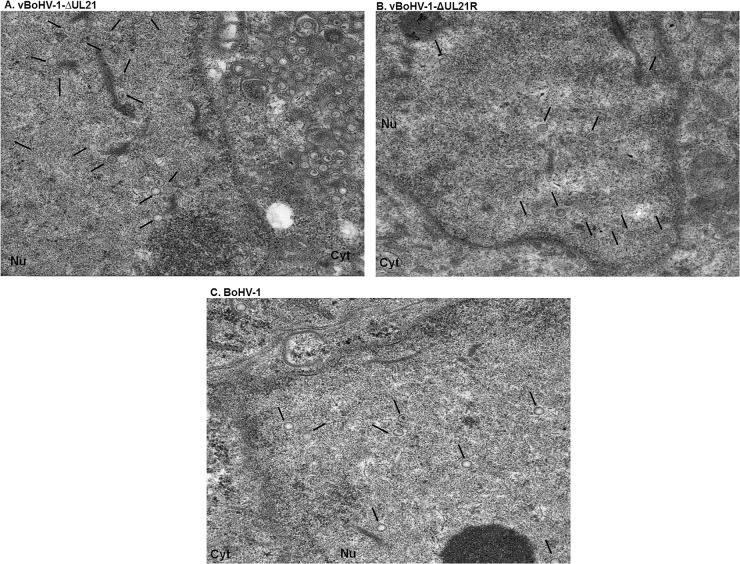
TEM micrographs of nucleocapsids in nuclei MDBK cells infected with the indicated viruses at a MOI of 3. At 18 hpi, the cells were fixed, sectioned, and analyzed using TEM. Overviews of virus infected cells containing nucleocapsids in the nucleus **(A)** vBoHV-1-∆UL21, **(B)** vBoHV-1-∆UL21R, **(C)** BoHV-1, showing no difference. Black arrows point to nucleocapsids.

To corroborate the fluorescence microscopy data, a TEM analysis was performed. MDBK cells were infected with the BoHV-1, vBoHV-1∆UL21, or vBoHV-1-∆UL21R viruses at a MOI of 3. The TEM analysis showed that nucleocapsid formation and movement from the nuclei to the cytoplasm did not appear to differ among the cells infected with the vBoHV-1∆UL21 (Figure 6A), vBoHV-1-∆UL21R (Figure 6B), or BoHV-1(Figure 6C) viruses. Furthermore, nucleocapsids quantification showed no significant difference (*p* > 0.05) in the total number of nucleocapsids in the nuclei infected with BoHV-1, vBoHV-1∆UL21, or vBoHV-1-∆UL21R viruses as shown in Figure 8 panel A. These findings showed that primary envelopment and nuclear egress, which was defined by capsid movement from the nuclei to the cytoplasm, remained unaffected, and that the differential distribution of capsids in the cytoplasm of cells infected with the *UL21* mutant likely resulted from impairment in secondary envelopment.

**Figure 7:**
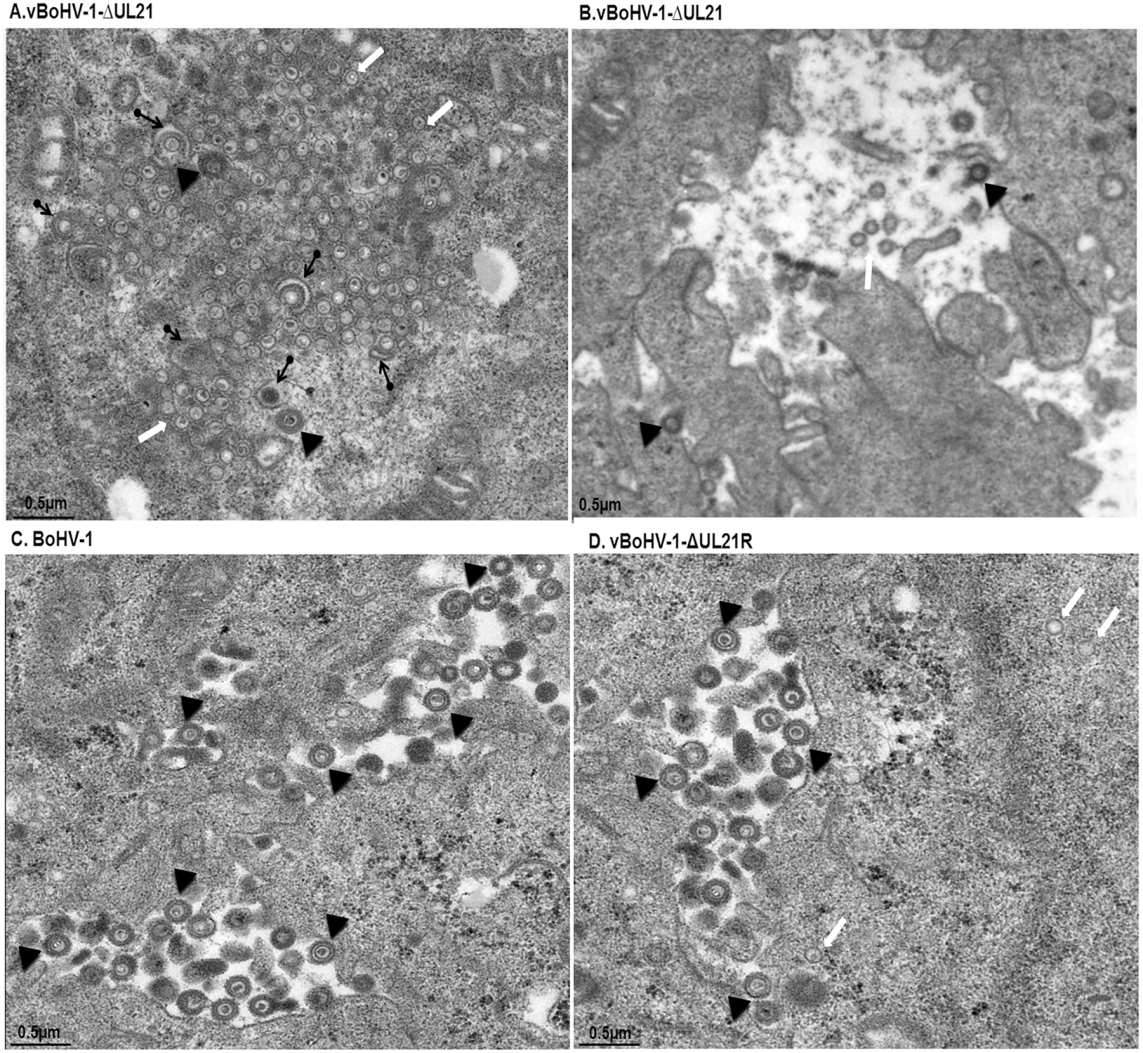
The vBoHV-1-ΔUL21 virus has a defect in secondary envelopment MDBK cells were infected with the vBoHV-1-∆UL21 (**A** and **B**), BoHV-1 **(C)**, or vBoHV-1-∆UL21R viruses **(D)** at a MOI of 3. At 18 hpi, the cells were fixed, sectioned, and analyzed by TEM. Cytoplasmic capsid clusters without secondary envelopment were observed in vBoHV-1-∆UL21-infected cells (A). In the vBoHV-1-∆UL21-infected cells, mature, virions were rarely seen in the cytoplasm and outer cell surface (B), compared with BoHV-1- (C) and vBoHV-1-∆UL21R-infected cells (D). White arrows point to aggregates of unenveloped cytoplasmic capsids, while arrowheads point to enveloped virions and black arrows with rounded tails point to partially enveloped virions.

**Figure 8:**
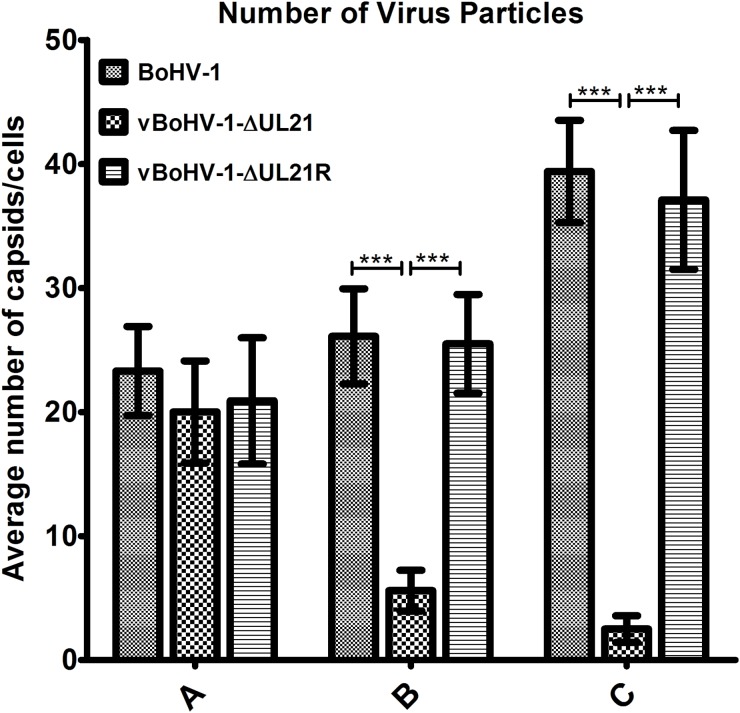
Quantitation of the virion distribution within cells using TEM MDBK cells were infected with the BoHV-1, vBoHV-1-∆UL21, or vBoHV-1-∆UL21R viruses at a MOI of 3. At 18 hpi, the number of virus particles in each well was calculated for 40 infected cells for each individual sample. *** indicates a statistically significant difference (*p* < 0.001). Bars indicate the mean values for each sample resulting from three independent experiments and error bars indicate the standard deviations. **(A)** Nucleocapsids in the nuclei. **(B)** Intracellular mature virions in the cytoplasm. **(C)** Mature virions on the cell surface.

### Effect of the *UL21* deletion on secondary envelopment

To analyze in more detail whether the replication defect of the vBoHV-1∆UL21 virus was associated with capsid assembly and maturation, MDBK cells were infected with the BoHV-1, vBoHV-1∆UL21, or vBoHV-1-∆UL21R viruses at a MOI of 3. At 18 hpi, the cells were processed and examined by TEM. The vBoHV-1∆UL21 virus exhibited a defect in secondary envelopment as evidenced by large numbers of capsid clusters that accumulated in the cytoplasm, which resulted in the failure of the capsids to become fully enveloped. Additionally, in vBoHV-1∆UL21-infected cells, many cytoplasmic capsids were observed to be partially enveloped or non-enveloped, while very few mature virions were observed in the cytoplasm (Figure 7A and 7B). No difference was observed in the growth between the BoHV-1 and vBoHV-1-∆UL21R viruses (Figure 7C and 7D), which confirmed that the defect in secondary envelopment and cytoplasmic exit was due to the deletion of the *UL21* gene. To quantify the effect of the *UL21* deletion, approximately 40 infected cells were counted in more than 25 fields in multiple sections of cells infected with the BoHV-1, vBoHV-1∆UL21, and vBoHV-1-∆UL21R viruses. Infected viral particles were categorized as nucleocapsids in the nuclei (A), intracellular mature virions (B), or extracellular enveloped virions (C). Although no significant difference (*p* > 0.05) was observed in the total number of nucleocapsids in the nuclei (Figure 8 panel A), the number of intracellular enveloped viral particles in the cytoplasm of the vBoHV-1∆UL21-infected cells was reduced by approximately 80% (Figure 8 panel B), while the number of extracellular mature virions was reduced by approximately 90% (Figure 8 panel C), compared with cells infected with the wild-type or vBoHV-1-∆UL21R viruses. Therefore, we concluded from the electron microscopic studies that *UL21* plays a critical role in secondary envelopment, as well as in the cytoplasmic exit of virions.

### Identification of the BoHV-1 *UL21*-interacting protein *UL16*

**Figure 9:**
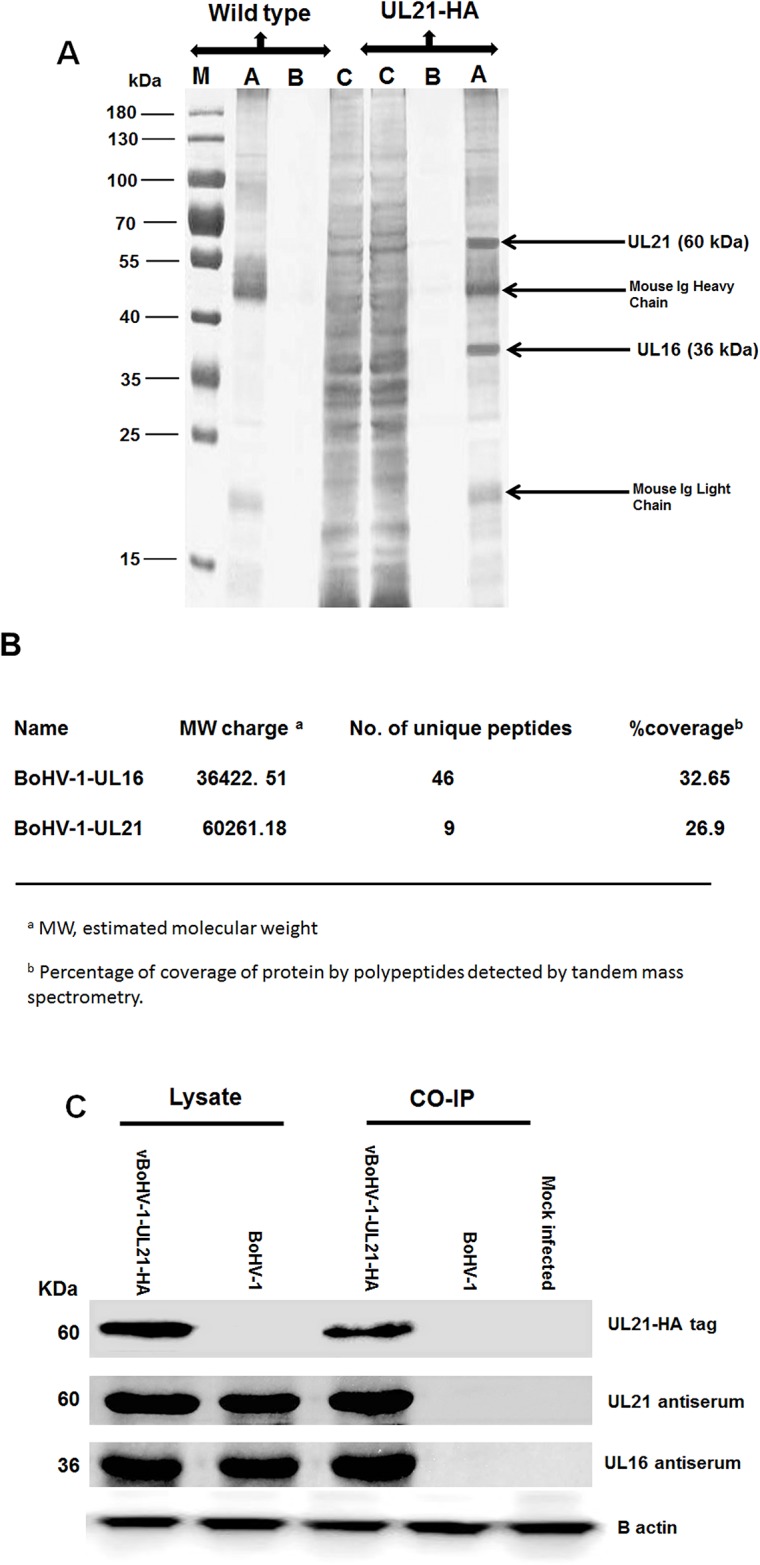
Characterization of the *UL21* protein by mass spectrometry **(A)** MDBK cells were infected with the vBoHV-1-UL21-HA or BoHV-1 viruses at a MOI of 3. At 18 hpi, cells were harvested and immunoprecipitated with anti-HA antibody conjugated magnetic agarose beads, and the precipitated proteins were loaded onto a 12% SDS-polyacrylamide gel and silver stained. Lane A, the bead eluate; lane B, the third wash; and lane C, the flow-through. Protein molecular weight markers in kDa are indicated to the left of the gel. **(B)** Mass spectrometry analysis of immunoprecipitated proteins along with the interacting proteins that correspond to the proteins. The expected 60-kDa UL21-HA band, as were 36-kDa band, which were confirmed to be *UL16* by LC-MS **(C)** MDBK cells were infected with the vBoHV-1-UL21-HA or BoHV-1 viruses, and proteins were precipitated using anti-HA antibody-conjugated magnetic agarose beads. The beads were electrophoresed and transferred onto PVDF membranes. Then, the membranes were probed with a mouse anti-HA-tag antibody and with the antisera indicated on the right side of the figure. Beta-actin was used as the internal reference control. The migration positions of proteins are shown in kDa to the left of the gel.

To investigate the *UL21* interactive proteins, MDBK cells were infected with the vBoHV-1-UL21-HA or BoHV-1 viruses. Cells were lysed at 18 hpi, and incubated with anti-HA-tag monoclonal antibody-conjugated magnetic agarose beads. Following sodium dodecyl sulfate-polyacylamide gel electrophoresis (SDS-PAGE) and silver staining, in addition to the 60-kDa *UL21* protein, one differential band corresponding to a 36-kDa protein was detected consistently (Figure 9A). To identify the protein(s), this band was subjected to a Liquid chromatography-mass spectrometry (LC/MS) analysis. The results of the mass spectrometry analysis revealed that most of the data corresponded to peptides from *UL16* (32.65% coverage with a 36,422.51 Da molecular weight. The results also indicated the presence of peptides from *UL21* (26.9% coverage with a 60,261.18 Da molecular weight) (Figure 9B). The molecular weight is the estimated molecular weight of the peptides, which can be calculated based on their mass-to-charge (*m*/*z*) values. Additionally, the observed UL21-HA band exhibited the correct molecular weight of 60 kDa, as the approximate size of *UL21* is 59 kDa and the size of the HA tag is approximately 1 kDa which proves the correct insertion and function of HA-tag (Supplementary Materials 1 and 2).

To confirm that *UL16* form complexes with *UL21*, co-immunoprecipitation assays were performed with either BoHV-1 or vBoHV-1-UL21-HA-infected cell lysates. Polyclonal antisera against GST-UL16 and GST-UL21 interacted with the immunoprecipitated lysates of the vBoHV1-UL21-HA virus, and 36-kDa (*UL16*) and 60 kDa (*UL21*) bands were detected in the lysates of vBoHV1-UL21-HA- and BoHV-1-infected cells (Figure 9C). However, no specific proteins were co-immunoprecipitated in lysates of BoHV-1-infected and mock-infected cells. The co-immunoprecipitation and immunoblotting analyses confirmed the mass spectrometry findings that *UL21* interacts with *UL16.*

### Observations of complexes of *UL21* with *UL16*

**Figure 10:**
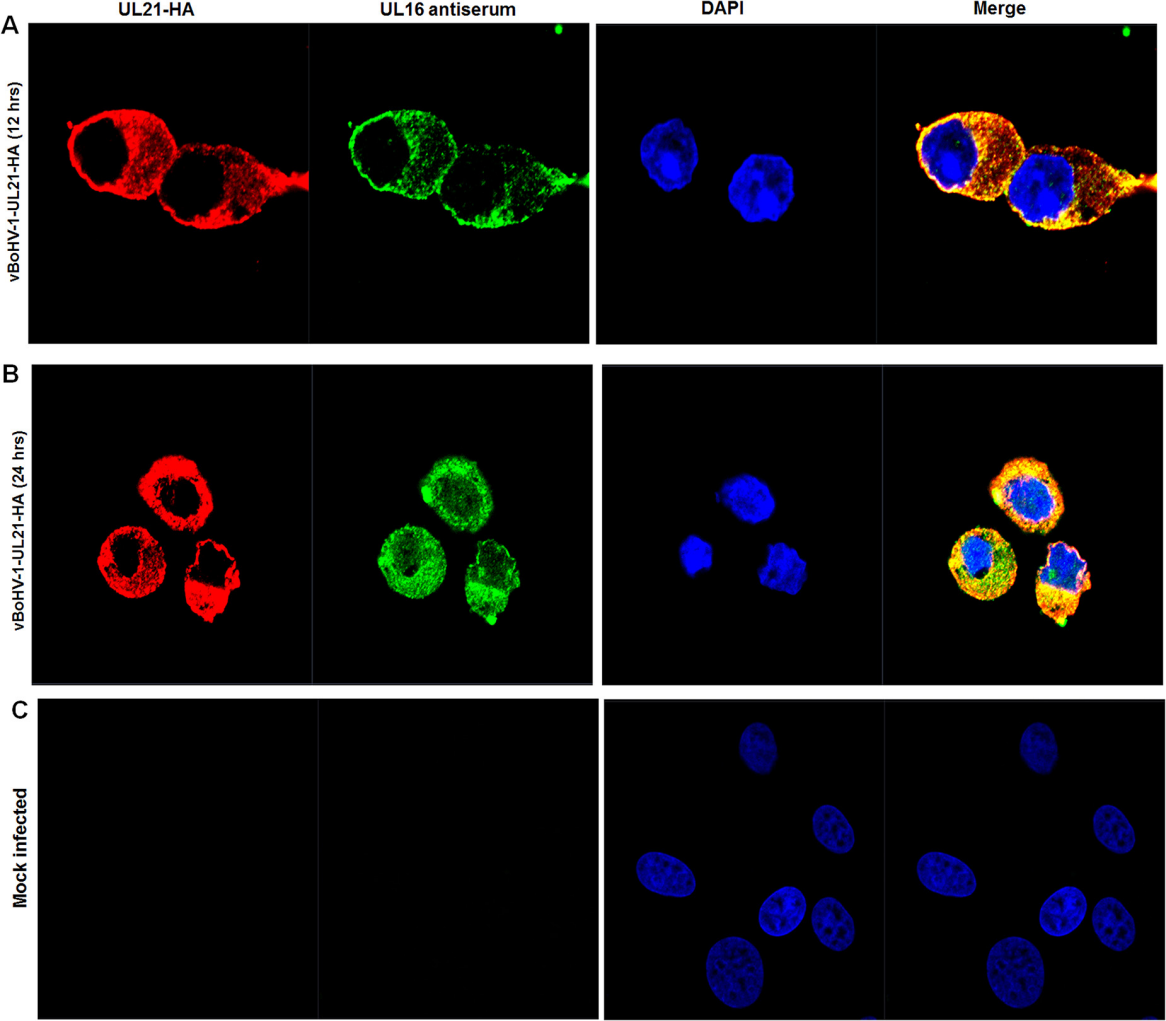
Interaction of *UL21* with *UL16* MDBK cells were infected with the vBoHV-1-UL21-HA virus at a MOI of 3, fixed for immunofluorescence and stained with a mouse anti-HA antibody or rabbit polyclonal antisera against *UL16* and Cy3-conjugated goat anti-mouse IgG or fluorescein isothiocyanate-conjugated goat anti-rabbit IgG antibodies. DNA was stained blue with DAPI. Fluorescence was observed under a Zeiss LSM 880 laser-scanning confocal microscope. **(A)** The *UL21* interaction with *UL16* at 12 hpi **(B)** 24 hpi **(C)** Mock-infected cells.

To confirm the interaction between *UL21* and *UL16*, we performed a co- immunofluorescence assay by using the vBoHV-1-UL21-HA virus. MDBK cells were mock-infected or infected with the vBoHV1-UL21-HA virus at a MOI of 3, fixed at 12 and 24 hpi, permeabilized, and stained with polyclonal rabbit antisera against *UL16* and *UL21*, as well as an anti-HA-tag monoclonal antibody. Confocal microscopy images showed that in the vBoHV1-UL21-HA-infected cells, *UL21* co-localized completely with *UL16* (Figure 10A and 10B). These data, combined with the co-immunoprecipitation results displayed in Figure 9C, indicated that *UL21* interacted with *UL16.*

## DISCUSSION

The functions of *UL21* in the life cycle of HSV-1, HSV-2, and PRV have been investigated extensively [10, 12–14, 23], whereas the functions of this conserved protein in BoHV-1 have not been well defined. In the present study, we constructed a *UL21* deletion mutant using a BAC system, which revealed the functions of *UL21* in BoHV-1 secondary envelopment and cytoplasm exit.

### BoHV-1 UL21 is required for efficient virus growth

Other studies have demonstrated that deleting *UL21* in HSV-1 [12, 23] and PRV [13, 14] viruses reduced both extracellular and intracellular virus yield and plaque size. The current study confirmed that *UL21* in BoHV-1 plays a similar important role in viral growth in cell culture. The deletion of *UL21* led to reduced virus titers which were 1,000 and 100-fold lower in the extracellular and intracellular environments at 48 hpi, respectively, compared with that of the wild-type virus, while the plaque size of the vBoHV-1-∆UL21 virus was 85% smaller than that of the wild-type, vBoHV-1-∆UL21R, and vBoHV-1-UL21-HA viruses. However, the extent to which UL21 mediates viral growth may be species-dependent. For example, unlike BoHV-1, an HSV-2 *UL21* mutant was unable to replicate until the *UL21* gene was restored to its original location [10]. These findings demonstrate that the *UL21* tegument protein is indispensable for efficient virus replication in cell culture, as deleting the *UL21* gene resulted in severe growth defects and smaller plaques compared with the BoHV-1, vBoHV-1-∆UL21R and vBoHV-1-UL21-HA viruses.

### BoHV-1 UL21 is required for secondary envelopment of capsids

In cell culture, we first discovered that in vBoHV-1-∆UL21-infected cells, the extracellular viral titer was lower than the intracellular viral titer. This showed that deleting *UL21* might impair the cytoplasmic exit of BoHV-1. By labeling *UL21* with an HA-tag, which was subsequently probed by an anti-HA-tag antibody and anti-UL21 serum, an immunofluorescence analysis showed that *UL21* localized predominantly to the cytoplasm, and it only exhibited a minor punctate nuclear staining. Our findings that *UL21* localizes to the cytoplasm and nucleus agree with previous studies of HSV-1, PRV [18–21, 23], and HSV-2 [10].

To investigate the potential role of *UL21* in viral capsid assembly and movement, HA-tagged viruses were studied with confocal microscopy and TEM. Both methods demonstrated that deleting *UL21* did not impair capsid movement from the nuclei to the cytoplasm, but it did affect cytoplasmic maturation and viral particle exit to the extracellular space. A similar association with nucleocapsids egress has been observed in HSV-1 and PRV UL21 [12, 14, 18, 23, 25]. However, in a previous study, HSV-2 UL21 was reported to be involved in nuclear egress [10]. Further observations highlighted that the *UL21* deletion caused an accumulation of partially enveloped or non-enveloped virions in the cytoplasm, as well as a decrease in the number of mature virions. This phenomenon demonstrated that the vBoHV-1-∆UL21 virus exhibited a severe defect in secondary envelopment and, therefore, that *UL21* is critical for BoHV-1 secondary envelopment. These results explain the present findings of differential titers between extracellular and intracellular viruses, because significantly fewer viruses matured and exited from the cytoplasm of the vBoHV-1-∆UL21 virus. Similar findings were reported previously, indicating that *UL21* is involved in capsid maturation [18]. While some studies showed that in the absence of *UL21*, capsid maturation remained unaffected in the PRV [14]. Therefore, although the general function of *UL21* in mediating viral morphogenesis of herpesviruses is conserved, its precise function varies in different viruses.

### BoHV-1-UL21 interacts with UL16

*UL16* is conserved tegument proteins in *Alphaherpesvirinae* [8]. In contrast to *UL21*, *UL16* has already been studied in some detail in PRV and HSV. In HSV-1 and PRV, deletion of *UL16* orthologs caused 10-fold reduction in virus propagation and defects in secondary envelopment [14, 26, 27]. Previously, in PRV the simultaneous absence of UL21 and UL16 led to capsid aggregations in the cytoplasm [14]. Currently in HSV-2 Δ16 mutant resulted in 200-950 fold reduction in virus propagation and suggested that *UL16* and *UL21* proteins may function together in nuclear egress [28]. *UL16* has the capacity to forms complexes with several viral proteins, including VP22, UL11, gE and UL21 [14, 17, 20, 21, 26]. So our recent studies strongly support this interaction between *UL21* and *UL16*.

In the present study, mass spectrometry analysis of proteins co-immunoprecipitated by HA-tagged *UL21* showed that *UL21* interacted *UL16*. Our immunoprecipitations observations reveal that the anti-UL16 serum efficiently co-precipitated the UL21 protein as previously reported by coimmunoprecipitation assays in PRV and HSV [14, 20]. Furthermore, an immunofluorescence microscopy analysis showed that *UL21* protein co-localized with *UL16* which was also observed in HSV-1 [21].

Unlike HSV-2, where *UL21* deletion prevented virus propagation and blocked nuclear egress [10], the absence of *UL21* in BoHV-1 did not affect nuclear egress. In HSV-1 [12, 15, 23], and PRV [13, 14], where *UL21* deletion mutants caused comparatively modest defects in virus growth while the *UL21* was found to be critical for BoHV-1 replication. In PRV UL21 mutant affected capsid maturation [18] and led to formation of DNA-deprived capsids [25], while in our study the *UL21* mutant affected the secondary envelopment. These results suggested that although the *UL21* is conserved among *alphaherpesviruses*, its precise function varies in different viruses.

Collectively, our results demonstrate that BoHV-1 UL21 plays critical roles in viral secondary envelopment and cell-to-cell spreading in tissue culture. Furthermore, we demonstrate that *UL16* forms a complex with *UL21* in BoHV-1. However, further research should be conducted to clarify whether the interaction of *UL16* with *UL21* correlates with *UL21* function and BoHV-1 lacking *UL16* would have a similar phenotype as BoHV-1 lacking *UL21*.

## MATERIALS AND METHODS

### Ethical statement regarding animal experiments

Experimental protocols involving animals were approved by the China Hubei Province Science and Technology Department, which is responsible for animal ethics (permit no. HZAURAB-2015-006), in accordance with the recommendations of the China Regulations for the Administration of Affairs Concerning Experimental Animals (1988) and the Hubei Regulations for the Administration of Affairs Concerning Experimental Animals (2005). The animal use in this study was supervised by the Ethical Committee for Experimental Animals of Huazhong Agricultural University, Wuhan, China.

### Cells and viruses

BoHV-1 mutant viruses (designated vBoHV-1) were derived from the BoHV-1 strain IBRV HB06 (which was isolated by our laboratory and stored as no. V201024 at the China Center for Type Culture Collection at Wuhan University, and used as the wild-type strain in the present study). BoHV-1 and mutant viruses were propagated and titrated in the Madin-Darby bovine kidney (MDBK) cell line as described previously [29]. Viral proteins were obtained from lysates of infected MDBK cells. Plasmid transfections of MDBK cells were conducted via the calcium chloride transfection method [30].

### Construction of *UL21* recombinant viruses

The *UL21* gene of BoHV-1 (GenBank accession no. AJ004801.1) encodes *UL21*, which comprises 575 amino acids. The procedures used to construct a *UL21* deletion mutant with a BoHV-1 bacterial artificial chromosome (BoHV-1 BAC) were described previously [31]. Briefly, the primers ΔUL21 F/R (Table 1), with 40-bp upper and lower homolog arms of the deleted region and sequences specific to the flanking sequences of a kanamycin resistance gene cassette, were used to polymerase chain reaction (PCR)-amplify the kanamycin resistance gene from the pEPkan-S plasmid, which was kindly provided by Dr. Nikolaus Osterrieder [32]. Then, the PCR products containing the upper and lower homologous arms of the deleted region and the positive selection marker of the 3′ I*-Sce*I-*aphAI* cassette, which includes AphAI conferring kanamycin resistance and an adjoining I-*Sce*I site, were purified using the Cycle Pure Kit (Omega Bio-Tek, Norcross, GA, USA) and confirmed by DNA sequencing. Subsequently, these gel-purified, *Dpn*I-digested, linear PCR amplicons were electroporated (1.8 kV, 25 μF capacitance, and 200 Ω resistance) into *Escherichia coli* GS1783 competent cells that contained a BoHV-1 BAC-positive clone (pBoHV-1 BAC). Chloramphenicol-resistant and kanamycin-sensitive colonies were identified and confirmed by PCR using the primers UL21-KAN-F/R (Table 1) and DNA sequencing, and they were named pBoHV-1-ΔUL21 BAC. Recombinant viruses were rescued by transfecting the respective BAC DNA into MDBK cells, and they were named vBoHV-1-∆UL21 (the Δ*UL21* mutant).

**Table 1:** List of primers used in this study

Primer name	Sequence (5’ to 3’)
**∆UL21-F**	TTGTTCCCCCCGTCGGCCCGCTCGCGCGGCCGCCGCGGCGGCTCAGCGTGCCACAAGGGCTAGGGATAACAGGGTAATCGATTT
**∆UL21-R**	TTATTGATTGCCGCGTCGCCGCCCTTGTGGCACGCTGAGCCGCCGCGGCCGCGCGAGGCCAGTGTTACAACCAATTAACC
**UL21-HA- F**	GCGAAGGGGAGGGAGAAGGGGAAGCTGGAGCCGGCACTGAACCCTACCCATACGACGTCCCAGACTACGCTTAGGCTCAGCGTGCCACATAGGGATAACAGGGTAATCGATTT
**UL21-HA -R**	TATTATTGATTGCCGCGTCGCCGCCCTTGTGGCACGCTGAGCCTAAGCGTAGTCTGGGACGTCGTATGGGTAGCCAGTGTTACAACCAATTAACC
**UL21-REPAIR-F**	TAAGGGTATATTATTGATTGCCGCGTCGCCGCCCTTGTGGCACGCTGAGCCTAGGGTTCAGTGCCGGCTC
**UL21-REPAIR-R**	TTTTGCGTCATTGTTCCCCCCGTCGGCCCGCTCGCGCGGCCGCCGCGGCGATGGAGCTCGCCTACCACAG
**UL21-KAN-F**	ACGCACCGCAGCAGCTCATC
**UL21-KAN-R**	CGCCAATAAAGACGCCTCCAA
**UL35-HA- F**	ATACGAGGAGGACGCTGGCGCGGCGGCGTCTGCCGCGCGCACGTACCCATACGACGTCCCAGACTACGCTTGACGCTTTTTTCGGTAGGGATAACAGGGTAATCGATTT
**UL35-HA -R**	ATTATTGCGATCGCACAATTGCGCCGATCCGAAAAAAGCGTCAAGCGTAGTCTGGGACGTCGTATGGGTAGCCAGTGTTACAACCAATTAACC
**UL35- F**	ATGTTTGCGACCTACGACCACG
**UL35- R**	CGCACAATTGCGCCGATCC
**cDNA UL21- F**	CGTGTACGCGCCCAACCGGGAGTGCTT
**cDNA UL21- R**	TGAACCCGTAGTCCGCCACGGATTCG
**GAPDH- F**	GGCCTGAACCACGAGAAGTATAA
**GAPDH- R**	CCCTCCACGATGCCAAAGT
**UL21-F**	GCGGATCCATGGAGCTCGCCTACC
**UL21-R**	CGAAGCTTCTAGGGTTCAGTGCCGG
**UL16-F**	GCGGATCC ATGGCCGAGGACCCCGCCGCTGCGG
**UL16-R**	CGAAGCTT TCAGCCCCACGGCGAGCCGCGCGCT

### Reversion of pBoHV-1-Δ*UL21*

A repair virus (vBoHV-1-∆UL21R) was constructed by restoring the entire *UL21* open reading frame (ORF) to its original location using the pBoHV-1-∆UL21 BAC (Figure 1C). The *UL21* coding sequence with flanking upstream and downstream sequences was amplified using the primers UL21-Repair F/R (Table 1). All the λ red recombination steps were performed according to a previous protocol [31], and positive clones were identified by PCR using the primers UL21-KAN-F/R (Table 1) and DNA sequencing. The *UL21* revertant virus vBoHV-1-∆UL21R was rescued by transfecting the respective pBoHV-1-∆UL21R BAC DNA into MDBK cells.

### Construction of hemagglutinin (HA)-tagged recombinant viruses

To investigate the effect of the *UL21* deletion, an HA tag was fused in frame with the sequence encoding the minor viral capsid protein UL35 (VP26) of the pBoHV-1 BAC (Figure 1E) and the pBoHV-1-∆UL21 BAC (Figure 1F) using the UL35-HA F/R primers (Table 1). The HA tag was also fused in frame with the sequence encoding the carboxyl-terminus of *UL21* in the pBoHV-1 BAC (Figure 1D) using the primers UL21-HA F/R (Table 1) via two-step, λ-red mediated mutagenesis [33]. All the two-step, λ-red recombination events were performed according to previously described methods [31], and positive clones were identified by PCR using the primers UL21-KAN-F/R, UL35- F/R (Table 1) and DNA sequencing. Recombinant viruses were rescued by transfecting the respective BAC DNA into MDBK cells, and the resultant mutants were designated as the vBoHV-1-UL35-HA, vBoHV-1-∆UL21-UL35-HA, and vBoHV-1-UL21-HA viruses.

### RNA extraction and reverse transcription-polymerase chain reaction (RT-PCR)

Freshly grown MDBK cells were infected with the indicated viruses at a MOI of 3 plaque-forming units (PFU) per cell. Total RNA was extracted from the cells using TRIzol reagent (Invitrogen, Auckland, New Zealand), and reverse transcription of cDNA was performed using the PrimeScript™ RT reagent Kit with gDNA Eraser (Takara, Otsu, Shiga, Japan) following the manufacturer’s instructions. The cDNA was quantified by RT-PCR with the specific primers cDNA-UL21 F/R (Table 1). The PCR products were sequenced by the Sangon Company (Wuhan, China).

### RFLPs

To further confirm the deletion in the recombinant mutants, genomic DNA was extracted from the mutant virus and the wild-type strain, and it was analyzed for RFLPs using the restriction enzyme *Hin*dIII (Takara, Dalian, China) as described previously [34]. After enzymatic digestion, the resulting DNA fragments were separated on a 0.7 % agarose gel at room temperature for approximately 15 h under a constant voltage of 20 V.

### Growth curve analysis

Virus replication kinetics was determined as follows. MDBK cells were infected with the vBoHV-1-∆UL21, vBoHV-1-∆UL21R, or wild-type BoHV-1 viruses at a MOI of 3 and incubated at 37°C for 2 h. Then, the inoculum was removed and infected cell monolayers were rinsed with low pH (3.0) citrate-buffered saline (40 mM citric acid, 135 mM NaCl, 10 mM KCl) for 1 min. The cells were washed twice with 1× phosphate-buffered saline (PBS) (136.9 mM NaCl, 2.7 mM KCl, 7.0 mM Na_3_PO_4_, and 0.9 mM Na_3_PO_4_ at pH 7.4), and fresh medium was added. Intracellular and extracellular viruses were harvested at 6, 12, 18, 24, 30, and 48 h. After three freeze-thaw cycles, 1000 RPM for 5 min centrifugation was used to clear the infected cell debris. The titers of the extracellular and intracellular viruses were determined by a plaque assay.

The plaque assay was performed in MDBK cells grown in 24-well plates. Serial dilutions of the viruses were prepared in Dulbecco's modified Eagle medium (Hyclone, Logan, UT, USA) supplemented with 1% fetal bovine serum (Gibco, Grand Island, NY, USA) and a 2% penicillin and streptomycin solution (Hyclone, Vienna, Austria) and inoculated into each well. After incubation for 2 h, the viral inoculum was removed, and the cells were overlaid with a 1:1 solution of carboxymethyl cellulose (3.2%) and Dulbecco's modified Eagle medium supplemented with 2% fetal bovine serum and 1% penicillin and streptomycin solution, and they were incubated for 48 h at 37°C in a 5% CO_2_ atmosphere. The cells were fixed at 48 h post-infection (hpi) with 4% paraformaldehyde and stained with crystal violet blue for 30 min. Plaque size was determined using an Olympus (Tokyo, Japan) IX70^®^ microscope.

To measure plaque size, confluent MDBK cells grown in a six-well culture plate (Corning, New York, NY, USA) were infected with 200 PFU of the indicated viruses in each well. For each virus, 50 plaques were measured microscopically in two independent experiments.

### Sodium dodecyl sulfate-polyacrylamide gel electrophoresis (SDS-PAGE) and western blotting

Approximately 8.8 ×10^8^ MDBK cells in a 100-mm petri dish were infected with the indicated viruses at a MOI of 3 for 18 h, and then the infected cells were washed and collected in 1× cold PBS with a cell scraper. Cells were collected and pelleted by centrifugation at 160 × *g* for 10 min at 4°C and lysed with lysis buffer (1% Triton X-100, 1 mM ethylenediaminetetraacetic acid, 50 mM Tris, and 150 mM NaCl) containing 1× protease inhibitor cocktail (Sigma-Aldrich, Shanghai, China). The proteins were separated by 15% SDS-PAGE and transferred to polyvinylidene fluoride (PVDF) membranes (Millipore, Hong Kong, China). The membranes were blocked overnight in 5% skim milk containing 0.1% Tween 20 in 1× PBS. Then, the membranes were probed with a mouse anti-HA-tag antibody (1:1,000 dilution; Beyotime, Haimen, China) or an antibody against β-actin as an internal reference (1:1,000 dilution; Beyotime). Goat anti-mouse antibodies conjugated with horse radish peroxidase (HRP) (1:5,000 dilution) (Southern Biotech, Birmingham, MI, USA) were overlaid for 90 min at room temperature. Then, the antibodies were removed, and the membranes were washed with Tris-buffered saline containing 0.1% Tween-20. The bound IgG was detected subsequently using the Super Signal West Pico Chemiluminescent Substrate (Thermo Fisher Scientific, Waltham, MA, USA).

### Silver staining

Silver staining was conducted as described previously [35]. Following electrophoresis, gels were fixed for 1 h in fixative solution (10% acetic acid, 40% ethanol, and 50% H_2_O), rinsed with distilled water overnight, and incubated in a 0.02% thiosulfate sodium solution for 1 min. After rinsing three times with water for 2 min, the gels were incubated in a 0.1% silver nitrate solution for 20 min at 4°C. The bands in the gel were developed in a 0.05% formaldehyde and 3% sodium carbonate solution, and the reaction was stopped by adding a 5% acetic acid solution for 5 min.

### Mass spectrometry

For the mass spectrometry analysis, a confluent monolayer of MDBK cells were was grown in 100-mm culture dishes and infected with the vBoHV1-UL21-HA and wild-type BoHV-1 viruses at a MOI of 3. At 18 hpi, the cells were lysed using lysis buffer. The cell lysates were added to magnetic agarose beads conjugated with an anti-HA-tag monoclonal antibody (MBL, Nagoya, Japan) and incubated for 5 to 6 h at 4°C with gentle rotation. Then, the agarose beads were washed five times, and protein complexes were eluted by heating them in 50 μl of 2× reducing SDS-PAGE sample buffer. The bound proteins were separated by SDS-PAGE and silver stained, and the expected band was excised and placed into a sterile 1.5-ml microcentrifuge tube for further processing via trypsin digestion for Liquid chromatography mass spectrometry (LC-MS), which was performed by Shanghai Applied Protein Technology, Shanghai, China.

### Immunofluorescence confocal microscopy

MDBK cells grown on glass coverslips were infected with the indicated viruses. At different time points, the cells were fixed in 4% paraformaldehyde at 37 C for 20 min, permeabilized with 0.2% Triton X-100, and blocked with 1% bovine serum albumen (Biosharp, Hefei, China) and 0.1% Tween 20 in PBS. The cells were incubated with mouse anti-HA antibodies (1:5,000 dilution; Beyotime) and then stained with Cy3-conjugated goat anti-mouse antibodies (1:1,000 dilution; Beyotime). Nuclei were stained blue with 4, 6-diamidino-2-phenylindole (DAPI) (Beyotime). After each step, the cells were washed three times with 1× PBS. Then, anti-fade mounting medium was used to mount the coverslips onto slides. The cells were examined, and images were captured with a Zeiss LSM 880 laser-scanning confocal microscope (Carl Zeiss, Jena, Germany).

### Transmission electron microscopy (TEM)

TEM was performed as described previously [31, 36]. MDBK cells were infected with the indicated viruses at a MOI of 3 PFU per cell. At 18 hpi, the cells were pelleted by centrifugation at 500 × *g* for 10 min at 4°C in cold PBS and fixed with 5% glutaraldehyde in 0.1 M sodium cacodylate buffer at 4°C for 2 h, and then they were post-fixed with 1% osmium tetroxide, followed by washing three times with PBS. The fixed cells were dehydrated with uranyl acetate and a graded ethanol series (35%, 50%, 70%, 90%, and 100%), polymerized using three changes of propylene oxide, embedded, sectioned, and examined by a transmission electron microscope (Hitachi 7000FA^®^, Tokyo, Japan).

### Protein expression and production of polyclonal antisera

Polyclonal antisera were produced according to a previously described method [37], with a slight modification. The entire coding sequences of the *UL16* and *UL21* were cloned by PCR amplification using the UL16 F/R, UL21F/R primer sets (Table 1), respectively, with KOD-Plus (Toyobo, Osaka, Japan). The PCR products were cloned into the prokaryotic expression vector pGEX-KG using the GST Gene Fusion System (Novagen, Darmstadt, Germany) via the *Bam*HI and *Hind*III restriction sites (Takara, Dalian, China). The cloned products were confirmed by DNA sequencing and named pGEX-16 and pGEX-21. The expression plasmids were transformed into *E. coli* strain BL21 (DE3) (TransGen Biotech, Beijing, China). Expression of all the glutathione S-transferase (GST) fusion proteins was induced using different concentrations of isopropyl-β-D-thiogalactopyranoside (*UL16*, 0.8 mM for 16 h at 37°C; *UL2*1, 0.8 mM for 4 h at 37°C). The bacterial cell pellets were collected by centrifugation (10,000 RPM/10 min), resuspended, and lysed by sonication. Then, the GST fusion proteins were separated from the lysate using Glutathione Sepharose 4B (GE Healthcare, Uppsala, Sweden). Subsequently, the purified proteins were used to immunize rabbits as described previously [38]. All the resulting polyclonal antibodies were used at a dilution of 1:3,000 (in 1% nonfat milk dissolved in 20 mM Tris, pH 7.6, 135 mM NaCl, and 0.1% Tween-20) for an immunoblotting assay, except that the rabbit polyclonal antiserum was diluted 1:100 for an immunofluorescence assay.

### Identification of UL21-interacting proteins

#### Co-immunoprecipitation assay for UL21-HA from infected MDBK cells

MDBK cells were seeded into 100-mm culture dishes and infected with the vBoHV-1-UL21-HA or wild-type BoHV-1 viruses at a MOI of 3, or mock infected as negative controls. At 18 hpi, the cells were lysed using lysis buffer. The cell lysates were added to magnetic agarose beads conjugated with an anti-HA-tag monoclonal antibody (MBL) and incubated for 5 to 6 h at 4°C with gentle rotation. Then, the agarose beads were washed five times, and protein complexes were eluted by heating the agarose in 50μl of 2× reducing SDS-PAGE sample buffer. For western blotting, the bound proteins were separated by 12% SDS-PAGE (Bio-Rad, Hercules, CA, USA) and transferred to a PVDV membrane (Millipore) that was blocked with 5% milk in PBS containing 1% Tween 20, and probed with polyclonal rabbit antisera against *UL16* and *UL21* (1:3,000 dilutions) and a mouse anti-HA-tag antibody (1:1,000 dilution, Beyotime) or with a β-actin antibody as an internal reference (1:1,000; Beyotime). Binding of the primary antibodies was detected using goat anti-rabbit and goat anti-mouse HRP-conjugated antibodies (1:5,000 dilutions; Southern Biotech, Birmingham, AL, USA). A chemiluminescent substrate (Thermo Fisher Scientific) was used to detect the proteins, and images were obtained using film.

#### Immunofluorescence assay

MDBK cells grown on 25-mm glass coverslips were infected with the vBoHV1-UL21-HA virus or mock infected. At 18 hpi, the cells were fixed in 4% paraformaldehyde, permeabilized with 0.2% Triton X-100, and blocked with 1% bovine serum albumen (Biosharp) and 0.1% Tween 20 in PBS. To detect the interactions of *UL16* with *UL21*, the cells were incubated with mouse anti-HA-tag antibodies (1:5,000 dilutions) or rabbit polyclonal antisera against *UL16* (1:100 dilutions) as indicated. Then, they were stained with Cy3-conjugated goat anti-mouse antibodies (1:1000 dilutions; Beyotime) or fluorescein isothiocyanate-conjugated goat anti-rabbit IgG antibodies (1:1000 dilutions; Beyotime). After each step, the cells were washed three times with 1× PBS. The nuclei were visualized by DAPI staining (Beyotime). Images were captured using a Zeiss LSM 880 laser-scanning confocal microscope.

### Statistical analysis

All experiments were performed independently at least three times. The data were expressed as means ± standard deviations, and the data from the various groups were compared using a *t*-test and two-way analysis of variance using GraphPad Prism software (GraphPad, La Jolla, CA, USA). Differences were considered to be statistically significant at *p* < 0.05 (*), and very significant at *p* < 0.01 (**) or *p* < 0.001 (***).

## SUPPLEMENTARY MATERIALS




