# Supplementary material for: Bovine herpesvirus 1 tegument protein UL21 plays critical roles in viral secondary envelopment and cell-to-cell spreading
Source: Oncotarget. 2017 Oct 10;8(55):94462–80. doi: 10.18632/oncotarget.21776 (PMC5706888; doi:10.18632/oncotarget.21776)
Supplement: Supplementary file 1 [file oncotarget-08-94462-s001.pdf]

Bovine herpesvirus 1 tegument protein *UL21* plays critical roles in viral secondary envelopment and cell-to-cell spreading

SUPPLEMENTARY MATERIALS

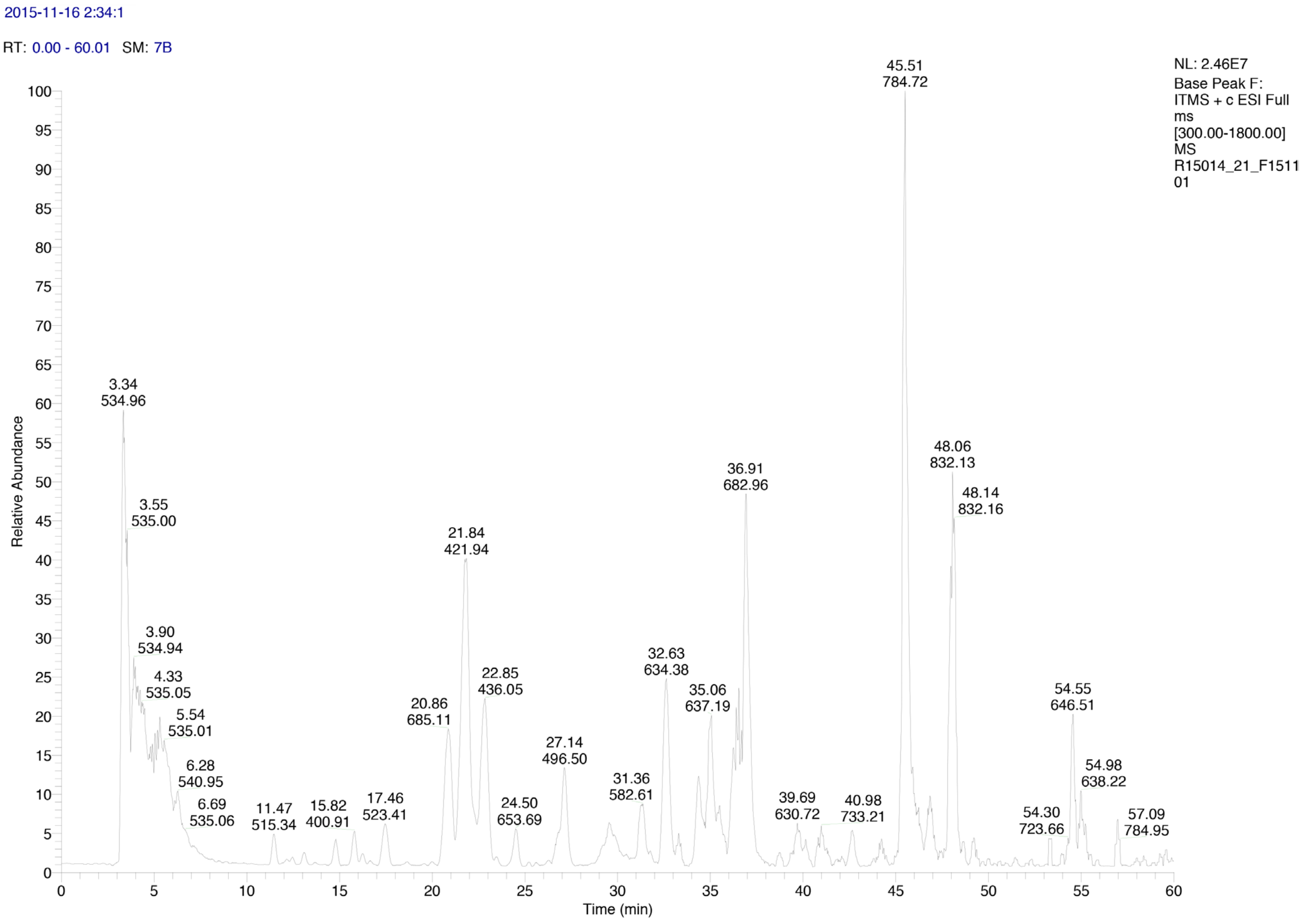

Supplementary Material 1: Liquid chromatography-mass spectrometry (LC/MS) analysis of immunoprecipitated proteins. Peptide mass spectra peak represents the tryptic peptide obtained from LC-MS analysis. The x axis indicates the time and the y axis represent the relative abundance. Molecular masses of the peptides calculated based on their mass-to-charge (*m/z*) values.

Supplementary Material 2: Liquid chromatography-mass spectrometry (LC/MS) analysis of immunoprecipitated proteins along with the interacting proteins that correspond to the proteins

See Supplementary File 1
